# Increased Tau Phosphorylation and Impaired Presynaptic Function in Hypertriglyceridemic ApoB-100 Transgenic Mice

**DOI:** 10.1371/journal.pone.0046007

**Published:** 2012-09-24

**Authors:** Nikolett Lénárt, Viktor Szegedi, Gábor Juhász, Aniko Kasztner, János Horváth, Erika Bereczki, Melinda E. Tóth, Botond Penke, Miklós Sántha

**Affiliations:** 1 Institute of Biochemistry, Biological Research Centre of the Hungarian Academy of Sciences, Szeged, Hungary; 2 Bay Zoltan Foundation for Applied Research, Institute for Plant Genomics, Human Biotechnology and Bioenergy, Szeged, Hungary; 3 Institute of Medical Chemistry, Faculty of Medicine, University of Szeged, Szeged, Hungary; Alexander Flemming Biomedical Sciences Research Center, Greece

## Abstract

**Aims:**

ApoB-100 is the major protein component of cholesterol- and triglyceride-rich LDL and VLDL lipoproteins in the serum. Previously, we generated and partially described transgenic mice overexpressing the human ApoB-100 protein. Here, we further characterize this transgenic strain in order to reveal a possible link between hypeprlipidemia and neurodegeneration.

**Methods and Results:**

We analyzed the serum and cerebral lipid profiles, tau phosphorylation patterns, amyloid plaque-formation, neuronal apoptosis and synaptic plasticity of young (3 month old), adult (6 month old) and aging (10–11 month old) transgenic mice. We show that ApoB-100 transgenic animals present i) elevated serum and cerebral levels of triglycerides and ApoB-100, ii) increased cerebral tau phosphorylation at phosphosites Ser^199^, Ser^199/202^, Ser^396^ and Ser^404^. Furthermore, we demonstrate, that tau hyperphosphorylation is accompanied by impaired presynaptic function, long-term potentiation and widespread hippocampal neuronal apoptosis.

**Conclusions:**

The results presented here indicate that elevated ApoB-100 level and the consequent chronic hypertriglyceridemia may lead to impaired neuronal function and neurodegeneration, possibly via hyperphosphorylation of tau protein. On account of their specific phenotype, ApoB-100 transgenic mice may be considered a versatile model of hyperlipidemia-induced age-related neurodegeneration.

## Introduction

Over the last few years, increasing number of evidence has highlighted the relationship between hyperlipidemia-induced cerebrovascular disorders and age-related cognitive decline [1]. Vascular dementia is a common cause of cognitive decline in the elderly and is associated with atherogenic dyslipidemia being specifically related to low density lipoprotein (LDL) and carotid atherosclerosis [2]. Elevated level of LDL leads to the development of atherogenesis and cardiovascular failure [3]. The major protein component of cholesterol- and triglyceride-rich LDL and VLDL lipoproteins is the apolipoprotein B-100 (ApoB-100), a 512 kDa lipoprotein. Recent studies indicate that ApoB-100-induced hyperlipidemia and atherosclerosis are not only implicated in the pathogenesis of cardiovascular disease but may also affect the cerebrovascular system [4] thus contribute to the development of neurodegenerative disorders [5]. Other studies have shown that Alzheimer's disease (AD) is accompanied by an elevation in apolipoprotein B concentration in the serum [6], [7]. AD represents the vast majority of all dementia cases. Most of AD cases are sporadic, less than 5% of cases of Alzheimer's disease (AD) can be traced to genetic causes, thus strengthening the hypothesis that lifestyle and nutrition-related factors may contribute to the development of metabolic syndrome and vascular lesions that eventually lead to neurodegenerative disorders [8].

The three major pathological hallmarks of AD are the extracellular deposition of amyloid beta, the formation of neurofibrillary tangles (NFT) and neuronal cell loss [9], [10], [11], [12]. Accumulation and plaque formation of Abeta peptide is the result of abnormal cleavage of amyloid precursor protein (APP) [10] and failure of amyloid clearance by enzymatic degradation, phagocytosis and cerebral perivascular transport [13], [14], [15]. Tau is a microtubule-associated phosphoprotein involved in microtubule assembly and stabilization. Hyperphosphorylation of tau leads to destabilization and disintegration of microtubular network and formation of neurofibrillary tangles (NFT) [11].

Previously, we generated a transgenic mouse strain overexpressing the human ApoB-100 protein [16]. On a standard diet, elevated serum triglyceride level, on cholesterol high diet an increased serum total cholesterol level and coronary atherosclerosis were observed [17]. High serum lipid level (hypertriglyceridemia and/or hypercholesterolemia) seriously affects the condition of cerebral arterial walls, gradually leading to a significant decrease in cortical microvascular density in ApoB-100 transgenic mice [4]. Cerebral microvascular lesions may cause: a) cerebral ischemia/hypoxia which in turn induces oxidative stress via the upregulation of NOS proteins; b) glucose-deprivation in cortical neurons, which may directly lead to apoptosis and neurodegeneration. It has also been shown that starvation induces tau hyperphosphorylation in the hippocampus and cortical tissues of mice [18].

The current work provides further insights into the interrelationship between hypertriglyceridemia and neurodegeneration. Using a previously generated transgenic mouse model we demonstrate here, that overexpression of human ApoB-100 leads to chronic hypertriglyceridemia, which in turn affects tau phosphorylation - without substantial amyloid accumulation-, eventually resulting in neurodegeneration and impaired long-term potentiation.

## Materials and Methods

### Animals

The study conformed to EU Directive 2010/63/EU and was approved by the regional Station for Animal Health and Food Control (Csongrad-county, Hungary) under Project License XVI/02752/2009. ApoB-100 transgenic mice were produced in our laboratory as described previously [16]. Transgenic mice were backcrossed with C57B/6 strain six times to achieve a homogenous genetic background. Young (3 month old), adult (6 month old) and aging (10–11 month old) mice were used in this study. Animals were maintained on a regular rodent chow diet. Animal surgeries were performed under sodium pentobarbital (Nembutal) anesthesia and all efforts were made to minimize pain and suffering.

### Measurement of serum triglyceride levels

Blood was collected from the tail vein of young and aging mice. Plasma total triglyceride levels were measured in triplicate according to the manufacturer's instructions using a commercially available enzymatic colorimetric assay kit (Diagnosticum Ltd., Budapest, Hungary). Test accuracy was monitored using Standard Lipid Controls (Diagnosticum Ltd., Budapest, Hungary), and colorimetric reactions were measured at 546 nm. Values were expressed in mmol/liter. Experimental groups (transgenic mice and wild-type littermates) consisted of 6 animals each.

### Lipid staining

The brains of adult and aging transgenic and wild-type mice were removed after transcardiac perfusion with isotonic saline. Tissues were postfixed with 4% formaldehyde-PBS solution for 1 h prior to embedding. Nile Red (Fluka) stain [19] was dissolved in acetone, and 10 µm acetone postfixed cryosections were stained at room temperature for 1 h in the dark [20]. Nuclei were counterstained using DAPI, and red and yellow-gold fluorescence was detected using a Nikon fluorescent microscope equipped with 590 nm and 528 nm filters, respectively. Cholesterol was stained with 0.05 mg/ml filipin-PBS solution at 37°C for 30 min in the dark (Filipin Complex was purchased from Sigma). Nuclei were counterstained with propidium iodide. Blue fluorescence was detected using a Nikon fluorescent microscope equipped with a 340–380 nm filter.

### Immunohistochemistry

Acetone-fixed brain cryosections (8–10 µm) from young and adult transgenic and wild type mice were prepared and incubated overnight with anti-ApoB-100 (Millipore, 1∶500), ApoE (Santa Cruz Biotech., 1∶100), and anti-pTauSer^404^ phosphospecific (Invitrogen, 1∶200) polyclonal antibodies. Sections were pretreated with 10 mg/ml collagenase type I (Sigma) for 10 min followed by an 1 hr incubation in 5% rabbit serum in PBS. Next, HRP-conjugated rabbit anti-goat or goat anti-rabbit secondary antibodies (Jackson Immuno Research) were added to the sections for 45 min at 1∶250 to 1∶800 dilutions. Peroxidase was detected using a reagent containing AEC (3-Amino-9-ethylcarbazole, Sigma).

Amyloid plaques were immunostained on brain sections of adult and aging mice using anti- Aβ [1–42] (Invitrogen, 1∶200) polyclonal antibody and Congo-Red (Sigma) staining.

### Conventional western blotting

The lipoprotein content of cortical brain lysates from adult transgenic and wild-type mice were evaluated using semi-quantitative western blotting. Samples containing 50 µg of total proteins were loaded onto a denaturing SDS-polyacrylamide gel, blotted to PVDF membrane, blocked with 3% BSA-TPBS or 5% non-fat milk-TPBS solution for 1 h and labeled with the corresponding primary antibody, specifically, anti-Apolipoprotein B-100 (Chemicon Int., 1∶500), anti-Apolipoprotein A-I (Rockland, 1∶500), anti-LDLr (Santa Cruz Biotech., 1∶100), anti-Apolipoprotein E (Santa Cruz Biotech., 1∶100), and anti β-actin (Sigma, 50 ng/ml) polyclonal antibodies, overnight at 4°C.To compare tau phosphorylation intensities in transgenic vs. wild-type mice the following site-specific primary antibodies were applied: anti-pTau Ser^199/202^ (Invitrogen, 1∶500), pTau Ser^262^ (Invitrogen, 1∶100), pTau Ser^396^ (Invitrogen, 1∶1000), pTau Ser^404^ (Invitrogen, 1∶3000). Total amount of tau protein was estimated using anti- TauV-20 antibody (Santa Cruz Biotech, 1∶100). APP content of young and adult mouse brains was determined using anti-APP N-terminal specific monoclonal antibody (Chemicon Int, 1∶1000). Then filters were washed and incubated with the corresponding secondary antibody (Jackson ImmunoResearch, at 1∶20,000–40,000 dilutions) for 45 min at RT. The signal was enhanced using Luminata Forte Western HRP Substrate (Millipore) and detected on Kodak X-ray film.

### Quantitative western blotting

Brain samples from adult transgenic mice and wild-type littermates were sent to Kinexus Bioinformatics (Vancouver, Canada) in order to monitor the tau and protein kinase Cγ (PKC γ) phosphorylation patterns. Tissue lysates were prepared, and western blottings were performed according to standard protocols (Kinetworks™ Immunoblotting Service) [21]. Separated mouse cerebral proteins were probed with 14 and 3 different primary antibodies specific for pTau (Ser^199^, Ser^199/202^, Ser^396^ and Ser^404^) and PKC γ (T^514^, T^655^ and T^674^) phosphosites, respectively, followed by an overnight incubation with specific secondary antibodies. Bands were visualized using enhanced chemiluminescense (ECL), and signals were collected using a highly sensitive 16-bit camera, detected with a Bio-Rad FluorS Max Multi-imager, normalized to time (60 seconds) and expressed as counts per minute (cpm) [21]. Phosphorylation of tau and PKC γ at specific sites in wild-type brains was classified as 100% baseline.

### Golgi-Cox staining

Aging wild-type (n = 4) and transgenic animals (n = 4) were sacrified by cervical dislocation, and the brains were removed. Golgi staining was performed using FD Rapid GolgiStain Kit (FD NeuroTechnologies) according to the manufacturer instructions. Briefly, brains were sliced into 10 mm blocks and were immersed into Golgi-Cox impregnation solution in the dark at room temperature for 3 weeks. Then 100 µm coronal sections were cut using a Microm HM 650 vibratome. Sections were collected in PBS and were mounted on gelatin-coated microscope slides.. Finally, sections were allowed to dry for 24 hours, then rinsed in distilled water for 4 minutes, After washing sections were dehydrated in 50%, 75% and 95% ethanol, for 4 minutes each, finally in absolute ethanol for 16 minutes. Dehydration was followed by clearing slides in xylene for 12 minutes, and sections were coverslipped with DPX mountant medium.

Dendritic spines were counted on neurons of stratum radiatum in the hippocampal CA1 region. Proximal apical dendritic areas were analysed at least 100 µm from the soma using a Zeiss light microscope. Digital photos were taken about second-oredered dendrites in 100 um length. Serial images were taken from each dendrites which were then stacked into one file. For counting dendritic spines we used the ImageJ 1,45 software. Dendritic spines were counted separately by 3 experimenters blind to the genotype.

### Neuronal apoptosis

Neuronal apoptosis was monitored in the hippocampal region of adult transgenic (n = 6) and wild-type mice (n = 6) using Fluoro-Jade C (Millipore) staining [22]. Staining was performed on 8–10 µm acetone fixed cryosections according to the manufacturer's protocol. Briefly, sections were dehydrated in descending alcohol series and dipped into KMNO_4_ solution for 30 min, then stained in 0.0004% Fluoro-Jade C (Millipore)-acetic acid solution for 30 min at room temperature protected from light. Stained sections were visualized using a fluorescent microscope (Nikon Eclipse E600) equipped with a 525-nm filter.

### Electrophysiological recordings

Hippocampal slices 350 µm in thickness were prepared from the brains of young and adult mice using a McIlwain tissue chopper (Campden Instruments, Loughborough, UK). Slices were incubated in standard artificial cerebrospinal fluid (ACSF) at room temperature for 60 min while constantly gassed with 95% O_2_, 5% CO_2_. ACSF was composed of 130 mM NaCl; 3.5 mM KCl; 2 mM CaCl_2_; 2 mM MgCl_2_; 0.96 mM NaH_2_PO_4_; 24 mM NaHCO_3_; 10 mM D-glucose, pH 7.4. Individual slices were transferred to a 3D-MEA chip with 60 tip-shaped and 60 µm high electrodes spaced by 200 µm (Ayanda Biosystems, S.A., Lausanne, Switzerland). The surrounding solution was quickly removed, and the slice was immobilized using a grid. The slice was continuously perfused with oxygenated ACSF (1.5 ml/min at 34°C) throughout the entire recording session. Data were recorded using a standard, commercially available MEA setup (Multi Channel Systems MCS GmbH, Reutlingen, Germany).

### Stimulation protocol

The Schaffer-collateral was stimulated by injecting a biphasic current waveform (−100/+100 µs) through one selected electrode at 0.033 Hz. Care was taken to place the stimulating electrode in the same region from one slice to the next. The peak-to-peak amplitudes of fEPSPs at the stratum pyramidale and stratum radiatum of CA1 were analyzed. After a 30-min incubation period, the threshold and maximum stimulation intensities for evoked responses were determined. To evoke responses, 30% of the maximal stimulation intensity was used. Following a stable 10-min control sequence, a paired-pulse protocol was applied. Briefly, two stimulation pulses were injected with interstimulus intervals of 50, 100 and 200 ms over 0.033 Hz. Three data points were obtained at every interstimulus interval.

### LTP and depotentiation protocol

Following a 10-min stable control sequence, LTP was induced using a theta-burst stimulation (TBS) protocol applied at the maximum stimulation intensity. TBS included four trains administered at 20 s intervals with 10 bursts at 5 Hz per train and 4 pulses given at 100 Hz per burst. LTP followed for 60 min, and depotentiation was the induced using low-frequency stimulation of 3 Hz for 5 min at maximum intensity. Depotentiated responses were followed for 30 min.

### Statistical analyses

Transgenic and wild-type groups were compared by one-way analysis of variance (ANOVA) followed by Bonferroni's post-hoc comparison and Student t-tests. Results were considered to be significantly different at a probability level of p<0.05. Data are presented as means ± SEM.

## Results

### Measurement of serum triglyceride levels

**Figure 1:**
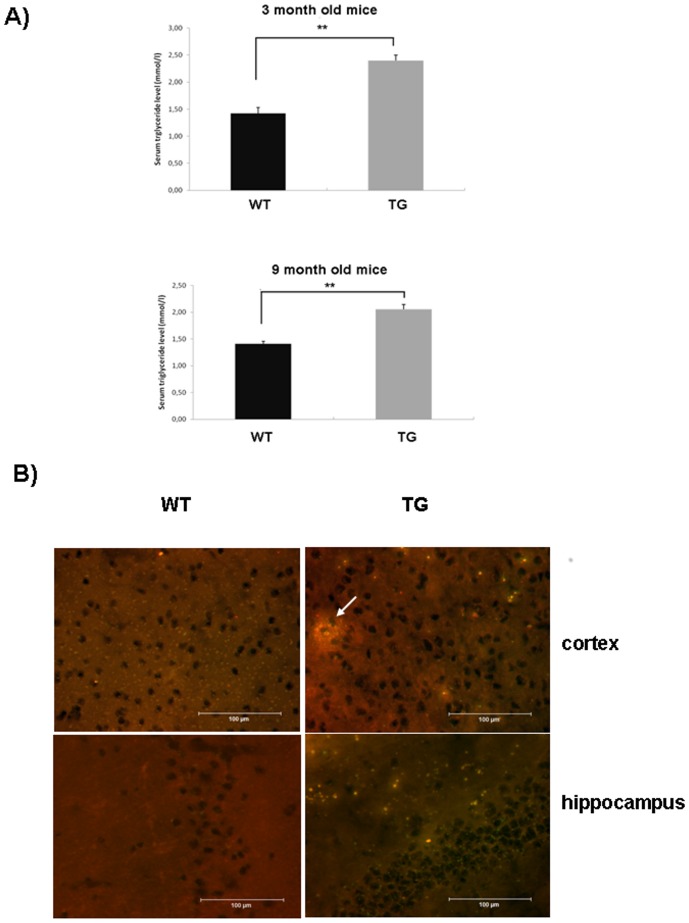
Detection of increased triglyceride levels in the serum and cerebral tissues of ApoB-100 transgenic mice. A) Serum triglyceride levels of 3- and 9-month-old wild-type (WT, n = 6) and ApoB-100 transgenic (TG) mice (n = 6). Values represent the mean triglyceride level (mmol/l) ± SEM, ** denotes statistical significant difference of p<0.01. B) Detection of triglycerides in the brain of 10-month-old wild-type (WT) and ApoB-100 transgenic mice (TG) using Nile-red staining (n = 3). Scale bars denotes 100 µm. White arrow indicates accumulation of triglycerides as lipid droplet in the cortex of aging transgenic mice.

ApoB-100 transgenic mice were generated in our laboratory as described previously [16]. We have previously demonstrated that human ApoB-100 protein is expressed at high levels in the liver and serum of transgenic mice [17], [23]. This time serum triglyceride levels in young and aging transgenic mice (n = 6) and their wild-type littermates (n = 6) were measured. ApoB-100 transgenic mice presented higher serum triglyceride levels (2.40 mmol/l±0.10) at the age of 3 months compared to their wild-type littermates (1.42 mmol/l±0.11), with a statistically significant difference of p<0.01. The difference in serum triglyceride levels persisted in aging transgenic mice as well, presenting a serum triglyceride level of 2.06 mmol/l±0.08 versus 1.40 mmol/l±0.05 in wild-type littermates, (p<0.01) (Figure 1A).

### Cerebral lipid metabolism

Next, we were interested in understanding whether chronically elevated serum triglyceride and ApoB-100 levels would affect cerebral lipid metabolism in transgenic mice. Consistent with the increased serum triglyceride level, cerebral triglycerides were markedly increased in cortical and hippocampal regions of aging transgenic mice revealed by Nile-red staining (Figure 1B). Moreover, in some cases triglyceride accumulation led to the formation of large cortical lipid droplets in aging transgenic mice (Figure 1B). However, no substantial changes were detected in the cerebral cholesterol level of aging ApoB-100 transgenics compared to wild-type littermates using filipin staining (Figure S1).

**Figure 2:**
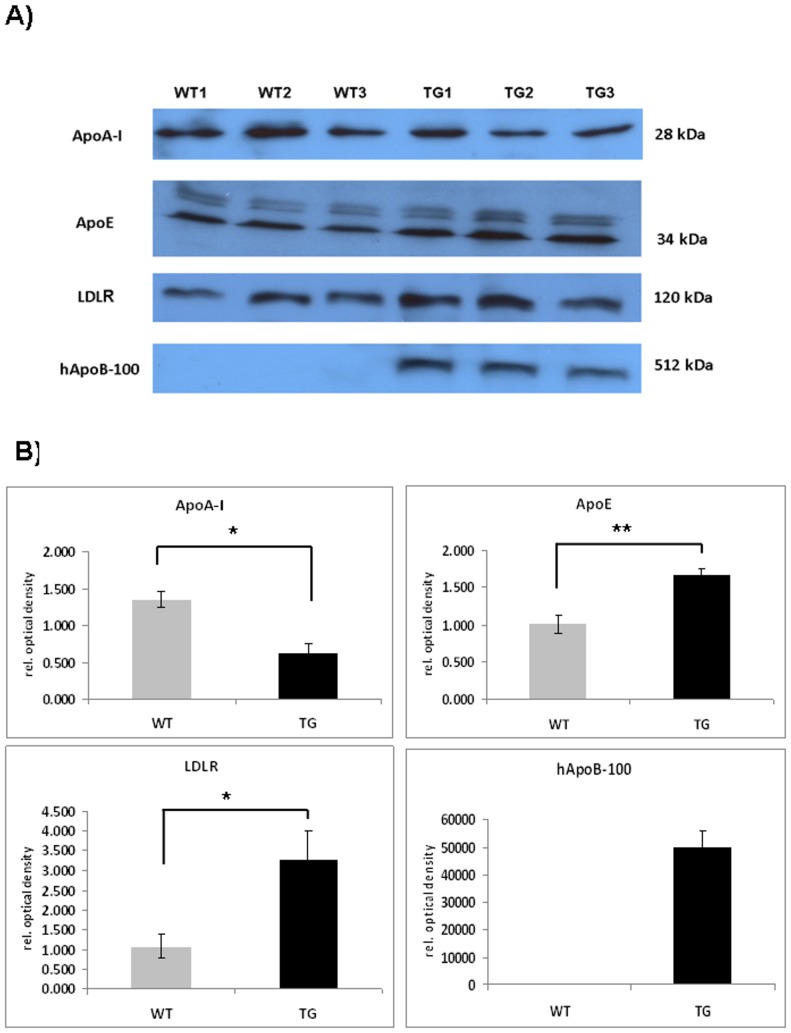
Cortical lipoprotein profile of hyperlipidemic transgenic mice. A) Detection of major apolipoprotein and LDLr levels in the cortex of adult (6 month old) wild-type (WT) (n = 3) and ApoB-100 transgenic (TG) mice (n = 3) using western blotting. B) Quantification of western blot experiments. Relative OD values were expressed as % of wild-type mice (100%), ± SEM, * denotes a statistical difference of p<0.05.

**Figure 3:**
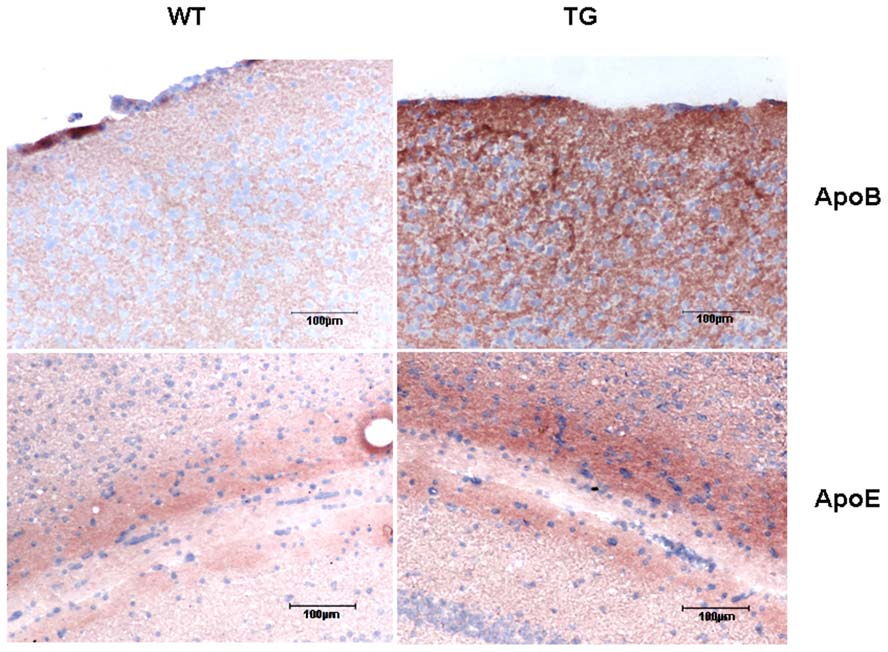
Increased expression of ApoB-100 and ApoE proteins in the cortical and hippocampal tissues of ApoB-100 transgenic (TG) mice. Immunohistochemical detection of ApoB-100 in the cortex (upper row) and ApoE in the hippocampal region (lower row) of adult wild-type (WT) and ApoB-100 transgenic (TG) animals. Magnification 200×. Scale bars represent 100 µm.

Next, we analyzed the expression of major apolipoproteins in the cortical region of adult ApoB-100 transgenics using western blotting. Suprisingly high level of ApoB-100 protein was detected in the cortex of adult transgenics while, the protein was not detectable in wild-type mice (Figure 2A). Cortical ApoE and LDL receptor levels were also significantly increased in transgenics compared to wild-type mice (p<0.01 and p<0.05, respectively). However, there was an apparent decrease in ApoA-I expression in the cortex of transgenic versus wild-type mice (p<0.05) (Figure 2A). Immunostainings confirmed increased level of ApoB-100 and ApoE proteins in the cortical and hippocampal regions of adult transgenic mice, respectively (Figure 3).

### Phosphorylation pattern of tau and PKCγ proteins

**Figure 4:**
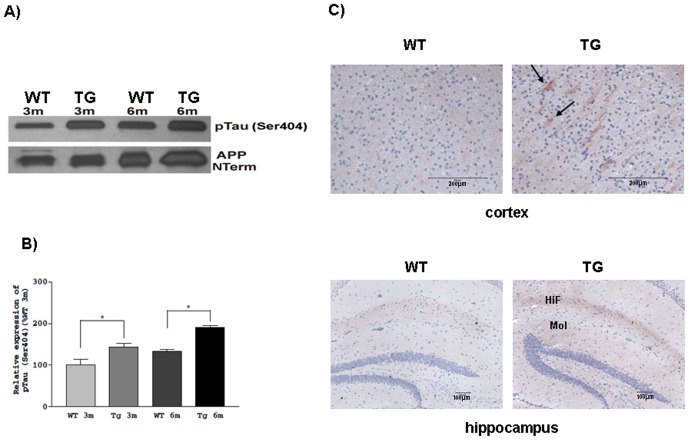
Increased expression of pTau Ser^404^ in transgenic animals. A) Representative picture of pTau Ser^404^ and amyloid precursor protein (APP) detection in young (3 month old) and adult (6-month-old) wild-type (WT) and ApoB-100 transgenic (TG) brain samples using western analysis B) Quantitative evaluation of western blots, (n = 3), ± SEM, * denotes statistically significant difference of p<0.05. C) Immunostaining of cortical and hippocampal regions from wild-type (WT) and ApoB-100 transgenic (TG) mice (n = 3) using anti-pTauSer^404^. Arrows indicate somatodendritic localization of hyperphosphorylated tau. HiF: hippocampal fissure; Mol: molecular layer of dentate gyrus. Scale bars represent 200 µm and 100 µm for the cortex and hippocampus, respectively.

**Figure 5:**
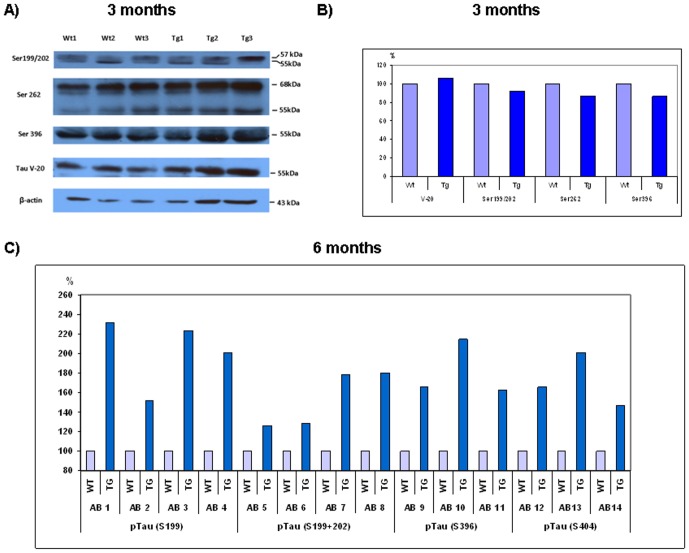
Increased tau phosphorylation in the brain of adult (6 month old) ApoB-100 transgenic mice. A) Tau phosphorylation intensities were compared in the cortex of young (3 month-old) wild-type (WT, n = 3) and ApoB-100 transgenic (TG) mice (n = 3), using conventional, semi-quantitative western blottings. B) Quantification of western blots. First, Tau V-20 was normalized to the endogeneous β-actin, then different phosphosites to Tau V-20. C) Quantitative western analysis of adult (6 month old) wild-type (WT) and transgenic (TG) brain lysates. Phosphorylation level at different tau sites in wild-type mice represents 100%.

Our next goal was to reveal the possible impact of chronic hypertriglyceridemia and elevated ApoB-100 level on tau phosphorylation. Hyperphosphorylation of tau protein is one of the key factors in the development of neurodegenerative diseases. Phosphorylation intensity of pTau at sites Ser^199^, Ser^199/202^, Ser^262^, Ser^396^ and Ser^404^ was investigated in young (3 month old) and adult (6 month old) transgenic mouse brains using semiquantitative- and quantitative-western blottings. Significantly increased phosphorylation of pTau Ser^404^ was detected in brain lysates of transgenic animals as young as 3 months old (p<0.05), and this phosphorylation was further increased in the adult animals (p<0.05) (Figure 4A and 4B). Hyperphosphorylated pTau Ser^404^ was localized to somatodendritic areas of the cortex and demonstrated enhanced staining in the hippocampus, specifically in the hippocampal fissure and molecular layer of the dentate gyrus (Figure 4C). Tau phosphorylation at Ser^199^/Ser^202^, Ser^262^ and Ser^396^ sites showed no substantial alterations in young transgenic animals versus wild-type mice (Figure 5A). Tau phosphorylation was further investigated by quantitative western analyses using 14 different commercially available antibodies specific for 4 different phosphosites of tau protein. Tau phosphorylation was considerably increased in transgenic mice, specifically at site Ser^199^ (with an average of 101.75%), Ser^396^ (with an average of 81.33%), Ser^404^ (with an average of 71.33%) and Ser^199/202^ (with an average of 53.25%) (Figure 5B).

### Amyloid plaque formation

**Figure 6:**
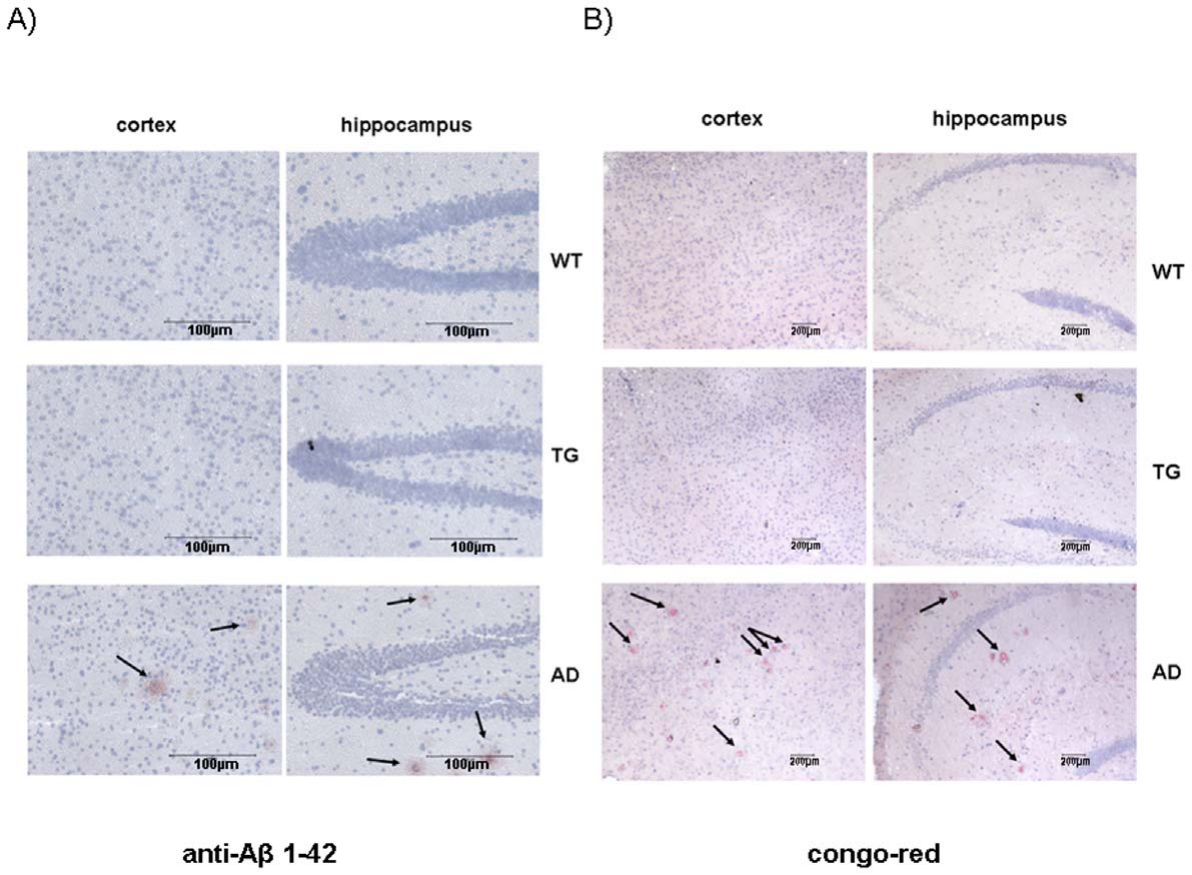
No amyloid plaques were detected in transgenic mice using immunohistochemistry. Immunohistochemical staining of β-amyloid plaques using A) anti-Aβ (1–42) polyclonal antibody B) congo-red staining on cerebral sections of aging (10 month old) wild-type (WT, n = 3), ApoB-100 transgenic (TG, n = 3), and AD model (APPSwe×PSEN1dE9) mice (n = 3). Nuclei were stained using hematoxylin stain. Arrows indicate stained amyloid plaques. Scale bars represent 100 µm (A) and 200 µm (B).

In addition to tau hyperphosphorylation, another pathological characteristic of Alzheimer's disease is the deposition of toxic Aβ peptides, aberrant cleavage products of amyloid precursor protein (APP), and formation of amyloid plaques in the brain. First, the expression level of APP in transgenic and wild-type brains was compared. APP level was increasing as the mice were getting older, with no difference between transgenic and wild type animals both at young or adult ages (Figure 4A). Amyloid plaque formation was further studied on brain slices of adult and aging animals by immunostaining using anti-Abeta (1–42) polyclonal antibody and Congo-Red staining. Brain sections from a widely used Alzheimer's disease mouse model (APPswe×PSEN1dE9) were used as positive control [24]. Although numerous amyloid plaques were detected on the cortical and hippocampal sections of AD mouse model, no plaque formation was observed on the corresponding brain sections of aging wild-type and transgenic mice (Figure 6A and 6B).

### Neuronal degeneration

**Figure 7:**
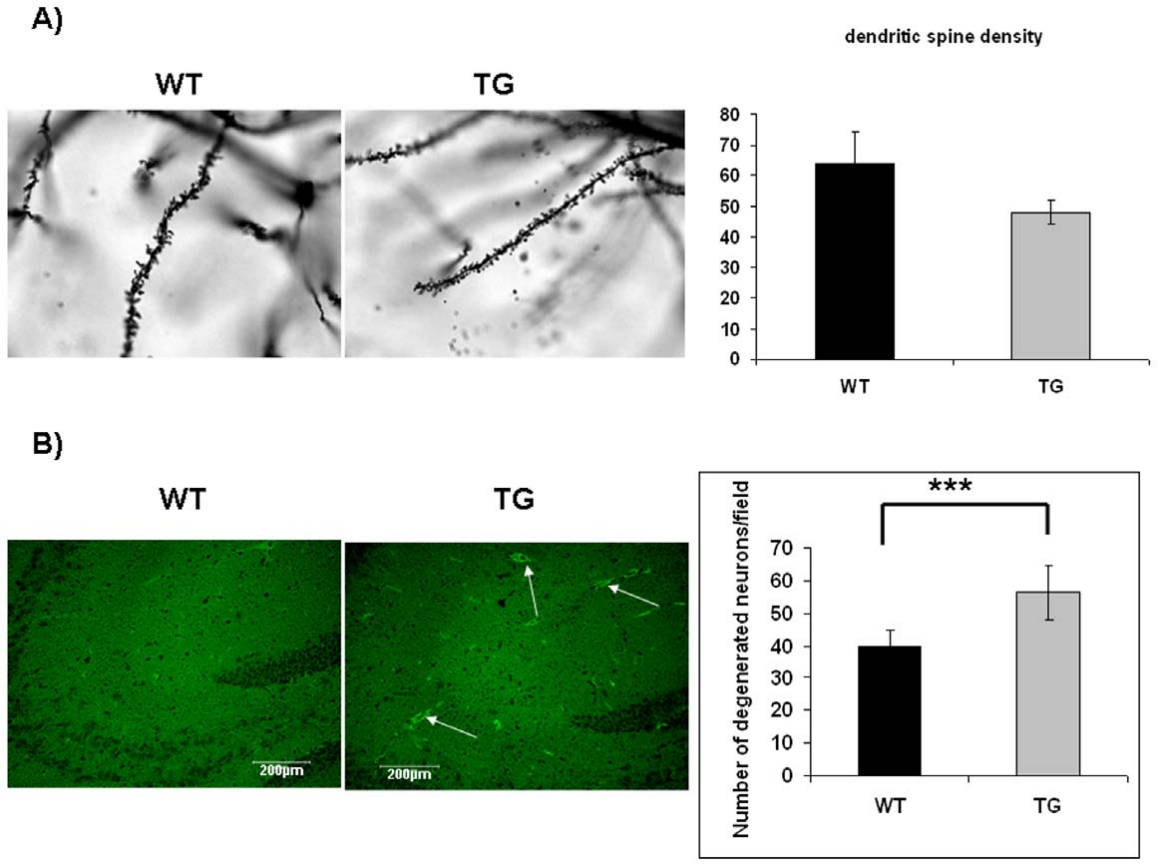
Increased neurodegeneration in ApoB-100 transgenic mice. A) Dendritic spine density in the hippocampus of aging wild-type (WT, n = 4) and transgenic (TG, n = 4) mice. Statistical analysis revealed a strong trend between genotypes (p<0.053). B) Detection of degenerated neurons in the hippocampal region of wild-type (WT) and ApoB-100 transgenic (TG) mice using Fluoro-Jade C staining (n = 6). Arrows indicate fluorescent degenerated neurons. Scale bars denote 200 µm. Quantification of degenerated neurons in the hippocampal region of wild-type (WT, n = 6) and ApoB-100 transgenic (TG) brains (n = 6), ± SEM, *** indicates a statistical difference of p<0.001.

Abnormal tau hyperphosphorylation results in disruption of axonal tubular network, which, in turn, leads to neuronal degeneration [11]. Neuronal degeneration was investigated by comparing dendritic spine densities in aging wild-type (n = 4) and transgenic mice (n = 4). Dendritic spines were counted on neurons of stratum radiatum in the hippocampal CA1 region after Golgi-Cox staining. Transgenic mice had shorter and fewer dendritic spines than wild-type controls (an average of 47.78±3.79 and 63.68±10.7, respectively) (Figure 7A). Although the difference between wild-type and transgenic groups was not statistically significant, however it showed a strong trend (p<0.053) (Figure 7A).

Neuronal apoptosis was studied using Fluoro-Jade C staining. This dye specifically stains degenerated neurons, independetly the mechanism of degeneration. Moreover, it not merely stains degenerated somas, but it is able to stain distal dendrites, axons and nerve terminals [22]. Degenerated neurons were counted in 5 microscopic fields (600 µm×600 µm) on hippocampal sections of transgenic (n = 6) and wild-type animals (n = 6). Significantly increased degenerated neurons were counted in the hippocampal region of adult transgenics versus wild-type mice (an average of 56.36±1.58 and 39.89±0.96, respectively) p<0.001 (Figure 7B).

### Electrophysiological recordings

**Figure 8:**
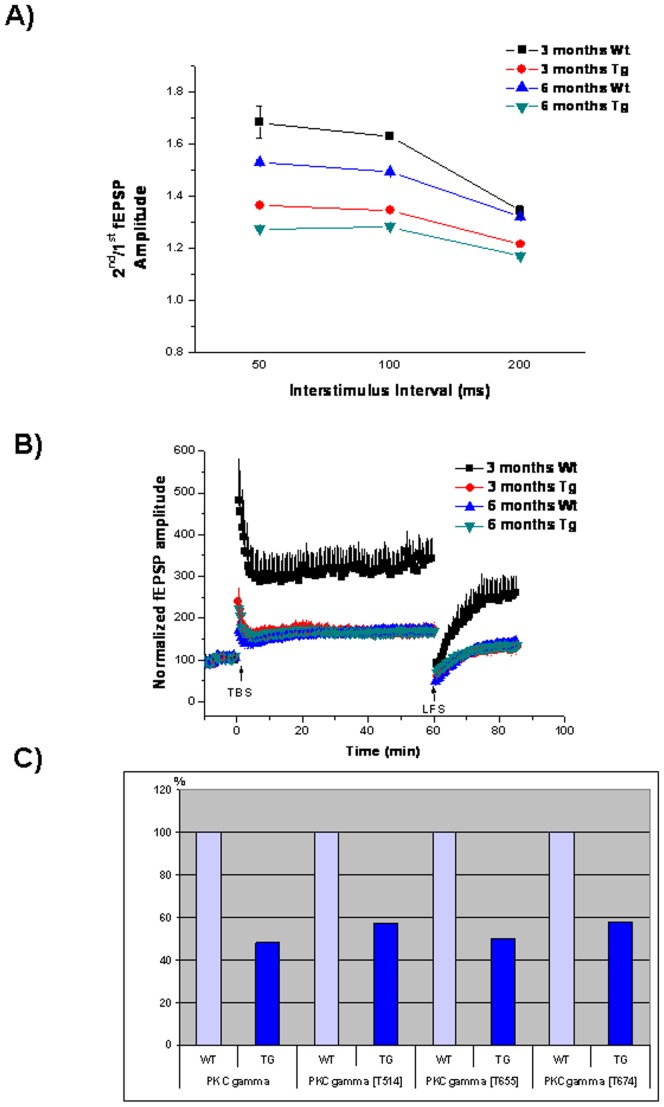
Impaired synaptic plasticity in hypertriglyceridemic mice. Electrophysiological recordings from brain slices of 3- and 6-month-old wild-type (Wt) and ApoB-100 transgenic (Tg) mice, (n = 6) A) Paired pulse facilitation (PPF), B) long-term synaptic plasticity. Arrows indicate theta burst stimulation (TBS) and low-frequency stimulation (LFS) C) detection of PKCγ expression in the brain of adult (6 month old) wild-type (WT) and transgenic mice (TG) using quantitative western blotting.

To determine whether biochemical and molecular biological changes in the brain of ApoB-100 transgenic animals influence presynaptic function, we performed. electrophysiological recordings on hippocampal slices of young and adult transgenic and wild-type mice. A total of 6 animals per group were tested, with an average of 5 electrodes recorded per slice. No significant differences were detected in the overall excitability of the slices (Figure 8A). A paired pulse protocol was applied with different interstimulus intervals (50, 100 and 200 ms). The amplitude of the 2^nd^ fEPSP is expected to be greater than that of the 1^st^ fEPSP in the CA1 region. This paired pulse facilitation (PPF) is used to assess presynaptic function. There were significant differences in the PPF ratio in apoB-100 transgenic mice compared to their wild-type littermates (Figure 8A). ApoB-100-overexpressing mice exhibited smaller PPF ratios at each interstimulus interval (1.35±0.01 at 50 ms, 1.34±0.01 at 100 ms and 1.21±0.01 at 200 ms at the age of 3 months, and 1.27±0.01 at 50 ms, 1.29±0.01 at 100 ms and 1.17±0.01 at 200 ms at 6 months) compared to wild-type littermates (1.68±0.062 at 50 ms, 1.63±0.01 at 100 ms and 1.35±0.02 at 200 ms at the age of 3 months, and 1.53±0.01 at 50 ms, 1.49±0.01 at 100 ms and 1.32±0.01 at 200 ms at 6 months; p<0.05 ANOVA, posthoc Bonferroni) (Figure 8A). In addition, aging itself affected presynaptic function, with adult mice exhibiting decreased PPF ratios compared to young mice, regardless of genotype (Figure 8A).

Long-term synaptic plasticity was measured by applying theta-burst stimulation to induce LTP. After 60 min, evoked responses were depotentiated using low-frequency stimulation (LFS). Slices from young ApoB-100 transgenic mice showed reduced LTP compared to wild-type slices [176±13% in transgenic mice and 343±46% in wild-type mice 60 min after theta-burst stimulation (TBS)] (Figure 8B). However, adult mice displayed the same level of potentiation regardless of genotype (168±10% in transgenic mice and 173±11% in wild-type mice 60 min after TBS) (Figure 8B). The rate of low frequency stimulation-induced depotentiation did not differ between the groups. When establishing the 15 min pre-LFS sequence as 100%, the amplitude of fEPSPs was 79±10% in young wild-type mice versus 75±12% in transgenic mice (Figure 8B). Thirty minutes after LFS, the amplitude of fEPSPs was 83±12% in adult wild-type mice versus 80±8% in transgenic mice (Figure 8B).

Next, expression of PKCγ protein and its phosphorylation intensity at T^514^, T^655^ and T^674^ phosphosites were investigated using quantitative western analyses. The expression of the protein was reduced by half and this change was reflected at every phosphosites (T^514^, T^655^ and T^674^) involved in the investigation (Figure 8C).

## Discussion

Previously, in our and other laboratories were also shown that overexpression of ApoB-100 in transgenic animals is accompanied by increased serum triglyceride levels [17], [25]. Under normal physiological conditions neither large serum lipoproteins (i.e ApoB-100) nor free cholesterol, triglycerides or other serum lipids are able to cross the blood-brain barrier (BBB). Nevertheless, we have detected high levels of plasma-derived ApoB-100 protein in brain tissues, indicating the dysfunction of the BBB, possibly due to chronic hyperlipidemia. Earlier findings are in agreement with our results and suggest that hypertriglyceridemia may contribute to endothelial dysfunction [26] likely through the generation of oxidative stress.

Here, we demonstrate that in hypertriglyceridemic transgenic mice tau is hyperphosphorylated at several sites and neurodegeneration develops in adult animals without notable β-amyloid deposition. Under normal circumstancies amyloid production and degradation/clearance is balanced. Alzheimer's disease is associated with impaired clearance of β-amyloid from the brain, a process normally facilitated by ApoE [27]. After acute treatment of APP/PSE1 mice with bexarotene, an RXR agonist and inducer of ApoE expression, rapid removal of both diffuse and compact Abeta plaques was observed [27]. In ApoB-100 transgenic mice we found that the production of β-amyloid – at least via APP- was not enhanced. On the other hand a substantial increase of ApoE expression in the cortex of ApoB transgenics was detected indicating an enhanced β-amyloid clearance. This result is in accordance with earlier report of Sparks et al. who demonstrated an increased density of cortical and hippocampal ApoE-immunoreactive neurons in rabbits fed a 2% cholesterol diet [28]. Other *in vitro* and *in vivo* studies also confirm that ApoE enhances cellular Abeta uptake and degradation [15], [29].

The binding of tau protein to microtubules is a phosphorylation dependent event, that is a result of equilibrium between tau kinases and phosphatases [30]. Hyperphosphorylation of tau has been demonstrated to increase dynamic instability, leading to disintegration of microtubular networks and eventually to formation of neurofibrillary tangles (NFT) [30], [31]. Antibodies recognizing pTau Ser^199^, Ser^202^, Se^262^, Ser^396^ and Ser^404^ are frequently used as AD diagnostic antibodies [32] indicating their important role in NTF formation in AD. A notable increase in tau phosphorylation was detected in adult ApoB-100 transgenic mice using these antibodies from different sources. Moreover, we observed an apparent increase in tau phosphorylation at phosphosite Ser^404^ in young (3 month old) transgenic mice.

In AD β-amyloid binds to and activates β_2_ adrenergic receptor signaling leading to subsequent activation of different serine and threonine protein kinases, which eventually result in enhanced tau phosphorylation [33]. In our transgenic strain tau hyperphosphorylation and neurodegeneration develop in the absence of amyloid plaques. Previous findings in transgenic mice overexpressing the familial AD mutant form of presenilin-1 (PS1) [34] or in conditional knockout PS1 mice generated on a PS2 null mutant background, show abnormal tau hyperphosphorylation, robust brain atrophy, impaired learning and memory and AD-like neurodegeneration in the absence of β-amyloid deposition [35]. However, the mechanism through ApoB-100 excess and hypertriglyceridemia can cause tau hyperphosphorylation remains to be elucidated. It has been previously shown that dietary and genetically-induced oxidative stress,- known to be present in our transgenic animals due to the impaired cortical microvascular density [4]-, may trigger tau hyperphosphorylation [36]. Another plausible explanation derives from the decreased arterial flow through the atherosclerotic vessels which in turn contributes to decreased glucose availability in the brain, a well known cause of tau hyperphosphorylation in the hippocampal and cortical tissues of mice [18].

It has been previously demonstrated that elevated serum triglyceride impairs NMDA-dependent hippocampal long-term synaptic potentiation, which may result in reduced hippocampal-dependent learning and memory [37]. Obesity and hypertriglyceridemia have also been shown to result in cognitive impairment that may be improved by a selective reduction in serum triglycerides [37]. In agreement with these findings we also recorded impaired presynaptic function and reduced long-term potentiation in hypertriglyceridemic transgenic mice very early, at 3 months of age. Pharmacological and electrophysiological studies have shown that several neuronal functions, including long term potentiation (LTP) and long term depression (LTD), specifically require PKC γ [38]. PKC γ deficient mice have modified long term potentiation (LTP) in the hippocampus, and exhibit mild deficits in spatial and contextual learning [39], [40]. In agreement with these previous results we have also found that PKC γ expression was decreased by half in the brain of adult ApoB-100 transgenic mice.

The interrelationship between hyperlipidemia/hypertrygliceridemia, cerebral vascular disorders and age related neurodegenerative disorders is a very plausible and discussed hypothesis. This study provides evidence that elevated ApoB-100 level accompanied by chronic hypertriglyceridemia can effect early on tau phosphorylation, and is linked to impaired neuronal function and widespread neuronal death in ApoB-100 transgenic mice. Based on the phenotype described above, our ApoB-100 transgenic model contributes to the understanding of hyperlipidemia-induced age-related neurodegeneration.

## Supporting Information

Figure S1 **Cerebral cholesterol level in wild-type and ApoB-100 transgenic mice.** Cholesterol was stained in the cerebral tissues of 10 month old wild-type (WT, n = 3) and transgenic (TG) mice (n = 3) using filipin dye. Nuclei were counterstained with propidium iodide (PI) stain. Magnification 200×. Scale bars indicate 100 µm.
(TIF)
