# Supplementary figures and images for: Increased Tau Phosphorylation and Impaired Presynaptic Function in Hypertriglyceridemic ApoB-100 Transgenic Mice
Source: PLoS One. 2012 Sep 24;7(9):e46007. doi: 10.1371/journal.pone.0046007 (PMC3454377; doi:10.1371/journal.pone.0046007)

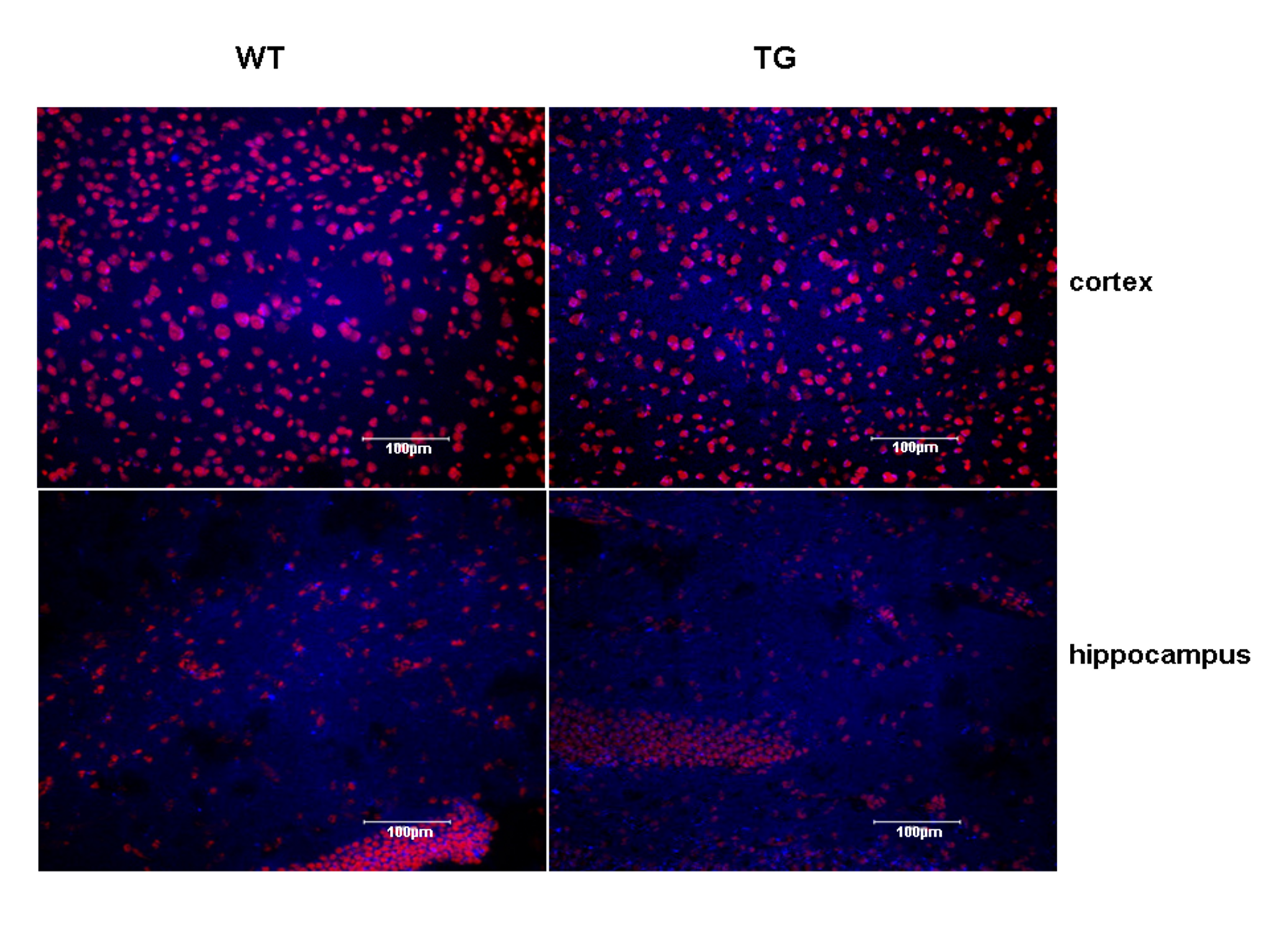

Supplement: Figure S1 — Cerebral cholesterol level in wild-type and ApoB-100 transgenic mice. Cholesterol was stained in the cerebral tissues of 10 month old wild-type (WT, n = 3) and transgenic (TG) mice (n = 3) using filipin dye. Nuclei were counterstained with propidium iodide (PI) stain. Magnification 200×. Scale bars indicate 100 µm. (TIF) [file pone.0046007.s001.tif]
